# The Response of the Gut Physiological Function and Microbiome of a Wild Freshwater Fish (*Megalobrama terminalis*) to Alterations in Reproductive Behavior

**DOI:** 10.3390/ijms25137425

**Published:** 2024-07-06

**Authors:** Yaqiu Liu, Chunni Kou, Jiayue Chen, Yuefei Li, Jie Li

**Affiliations:** 1 Pearl River Fisheries Research Institute, Chinese Academy of Fishery Sciences, Guangzhou 510380, China; 13239095043@163.com (C.K.); liyuefei815@163.com (Y.L.); 2 Guangzhou Scientific Observing and Experimental Station of National Fisheries Resources and Environment, Guangzhou 510380, China

**Keywords:** *Megalobrama terminalis*, reproduction, metagenome, interaction, digestive functionality

## Abstract

The fish gut microbiome is well known for its role in degrading nutrients to improve the host’s digestion and absorption efficiency. In this study, we focused on the core physiological adaptability during the various reproductive stages of the black Amur bream (*Megalobrama terminalis*) to explore the interaction mechanisms among the fish host gut mucosal structure, gut enzyme activity, and gut microbial metabolism in the course of the host’s reproductive cycle. Our findings showed that *M. terminalis* exhibited locomotion metabolic type (aids in sporting) in the reproductive stage, and a change to visceral metabolic type (aids in digestion) during non-reproductive and post-reproductive stage phases. The impact of metabolic type selection and energy demand during various reproductive stages on fish nutrition strategy and digestive function was substantial. Our resulted showed that mitochondria in intestinal epithelial cells of reproductive *M. terminalis* appeared autophagy phenomenon, and the digestive enzyme activities in the intestines of reproductive *M. terminalis* were lower than those in the non-reproductive and post-reproductive individuals. Moreover, these differences in nutrition strategy have a prominent impact on the gut microbiome of reproductive *M. terminalis*, compared to non-reproductive and post-reproductive samples. Our findings showed that reproductive females had lower levels of alpha diversity compared to non-reproductive and post-reproductive females. Our results also showed a greater functional variety and an increase in functional genes related to carbohydrate, lipid, amino acid, cofactors, and vitamin metabolic pathways in the NRS and PRS group. It is noteworthy that an enrichment of genes encoding putative enzymes implicated in the metabolism of taurine and hypotaurine was observed in the RS samples. Our findings illustrated that the stability and resilience of the gut bacterial community could be shaped in the wild fish host–microbiome interactions during reproductive life history.

## 1. Introduction

The status of the gut microecology is a valuable proxy for estimating the physiological states of host species and is essential for host survival [1,2,3,4]. The complex gut microecological structure can strengthen the metabolic capacity of the host and provide a series of benefits such as maintaining the digestive capacity for nutrients, regulating the balance of energy metabolism, improving immune function, and resisting the invasion of pathogens [5,6,7]. As an important part of gut microecology, the gut microbiome can synthesize essential amino acids, vitamins, and short-chain fatty acids for the host, and assist the host in degrading plant polysaccharides and other nutrients to improve the host’s digestion and absorption efficiency [6]. Additionally, host behavioral processes such as predation and reproduction, which are considered to be important predictors of differences and similarities in the metabolic functions of the core gut microbiota, have been demonstrated to be affected by the gut microbiota in several species [8]. For example, changes in the community characteristics of mammalian intestinal core symbiotic microbiomes were significantly correlated with enzyme activities related to host nutrient metabolism [5]. The gut microbiome impacts host reproductive behavior by affecting the host’s nervous system and peripheral chemical communication [9]. Balanced interactions between host cells and non-host cells, including the local microbial community, are crucial for the survival and reproduction of the host [10].

Recent research indicates that there is bidirectional communication between the microbiome and the hypothalamus–pituitary axis, as well as the gut–brain axis, which can influence the host’s endocrine system significantly [10]. The microbiome significantly influences the female reproductive endocrine system by interacting with estrogen, androgens, and other hormones throughout a woman’s life [8]. A variation in reproductive hormones associated with pregnancy and lactation may drive differences in gut bacterial community between reproductive and non-reproductive rhinoceros females [11]. High levels of stress hormones are linked to alterations in the composition of gut bacteria, which can impact the reproductive success of female hosts [8]. Fish are the most varied group of vertebrates on earth, involving over 34,000 species, and possess a wide range of physiological and ecological adaptations. Fish typically have several bacterial symbionts in their intestines, which can impact their growth, reproduction, population dynamics, and susceptibility to disease. Reproductive behavior plays a crucial role in energy management and is closely linked to the gut microbiota [12]. Yet, there are fewer studies on the gut microbiome’s impact on fish reproductive behavior compared to mammals [9,13]. Most research on fish gut microbiota has focused on investigating the effects of nutritional supplements on growth, development, and health within the microbiome of commercially important or model fish species [14]. Existing research has shown that the specialized intestinal mucosal structure and intestinal core microbiota function can enhance the tolerance of wild fish to fluctuations in external resources and improve the efficiency of extracting nutrients from external foods [15,16,17]. Nevertheless, the causal nature of the association between specific microbes and fish host physiology remains unclear. How fish reproduction affects the fish gut microbiome thus requires further research.

The Pearl River is the third longest river in China, with a total length 2320 km, providing an important habitat for numerous economically and ecologically important fish species. The black Amur bream (*Megalobrama terminalis*) is a species with remarkable digestive flexibility that plays a significant role in fisheries productivity in the lower parts of the Pearl River [18,19]. Fish metabolic activities are influenced by their environment and behavior, particularly in response to varying water currents [20]. In our preceding research, the timing and intensity of river flooding were regarded as critical factors for the reproductive success of the fish species *M. terminalis* [21]. In habitats characterized by swift water flow, fishes often experience demands on their metabolism that push them to the limit of their aerobic capabilities, also known as their maximum metabolic rate [20]. Therefore, it is possible that *M. terminalis* faces a physiological tradeoff between ‘locomotion metabolic type’, which might sacrifice some digestive efficiency for the sake of improved movement capabilities, and ‘visceral metabolic type’, characterized by higher efficacy in digestion particularly during the fish’s reproductive life history.

Moreover, our previous study indicated that the gut microbiota of *M. terminalis* can undergo adaptive variation during migration, and this species is capable of altering its own gut microbial community and function in order to enhance the utilization efficiency of external food in various habitats [12,22]. Considering the generally close relation between the gut microbiome and the host, we hypothesized that there may be a potential interaction between the metabolic-type tradeoff of wild *M. terminalis* and variation in its gut microbiome during the reproductive life history. In the current study, we focused on the host gut mucosal structure, gut enzyme activity, and gut core microbial metabolism and explored the core physiological adaptability in different fish reproductive stages. The aims of the study were to elucidate the changes in the intestinal microecological structure and metabolic functions during the breeding period of wild *M. terminalis*, and to verify the proposed hypothesis. The research results will enrich the basic physiological and ecological knowledge concerning freshwater fish reproduction and will provide a scientific basis for the protection of fish reproductive activity in the wild.

## 2. Results

### 2.1. Biological, Gut Morphological and Physiological Responses in Different Reproductive Stages

**Figure 1:**
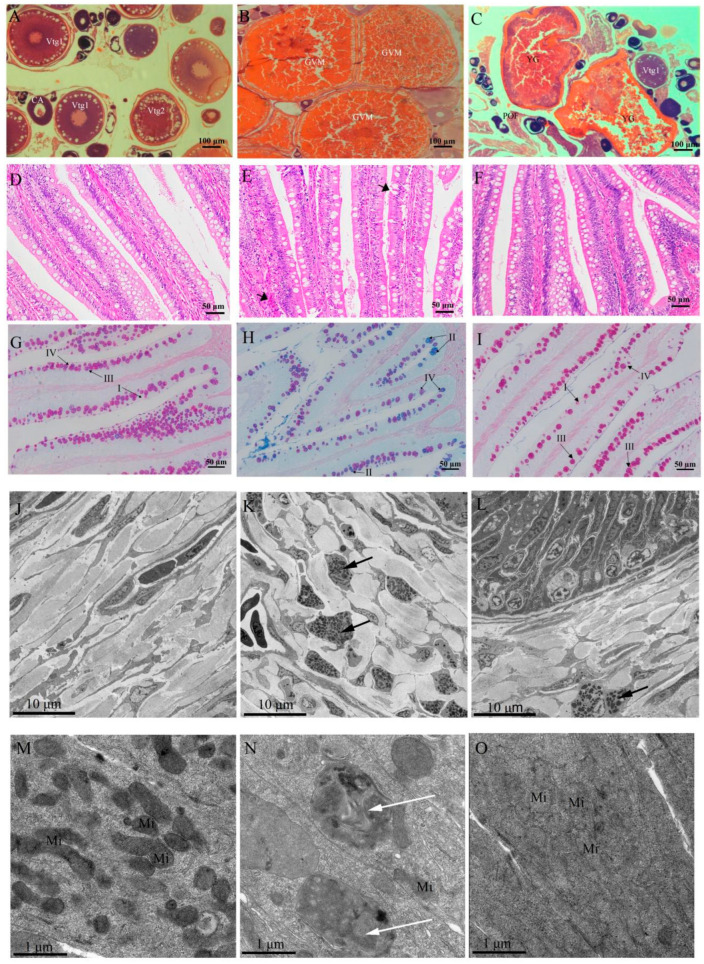
Morphological characteristics of ovary and gut of the black Amur bream in the different reproductive statuses. (**A**–**C**) Transverse sections of ovary of the black Amur bream in the different reproductive statuses ((**A**). non-reproductive stage; (**B**). reproductive stage; (**C**). post-reproductive stage); (**D**–**F**) Hematoxylin and eosin-stained images of intestinal tract of the black Amur bream in the different reproductive statuses ((**D**). non-reproductive stage; (**E**). reproductive stage; (**F**). post reproductive stage). (**G**–**I**) Mucous cell in the intestinal tract of the black Amur bream in the different reproductive statuses ((**G**). non-reproductive stage; (**H**). reproductive stage; (**I**). post reproductive stage); (**J**–**O**) Ultrastructural observation of intestinal eosinophilic granulocyte (**J**–**L**) and mucosal epithelial cell (**M**–**O**) of the black Amur bream in the different reproductive statuses. ((**J**,**M**). non-reproductive stage; (**K**,**N**). reproductive stage; (**L**,**O**). post-reproductive stage); note: GVM, germinal vesicle migration; POF, post-ovulatory follicle; Vtg1, primary vitellogenic oocyte; Vtg2, secondary vitellogenic oocyte; YG, yolk. Black arrow signs indicate damaged intestinal mucosa. I, type I mucous cell; II, type II; mucous cell; III, type III mucous cell; IV, type IV mucous cell; black arrow signs indicate eosinophilic granulocyte in intestinal submucosa. White arrow signs indicate mitochondria autophagy in intestinal epithelial cells.

**Figure 2:**
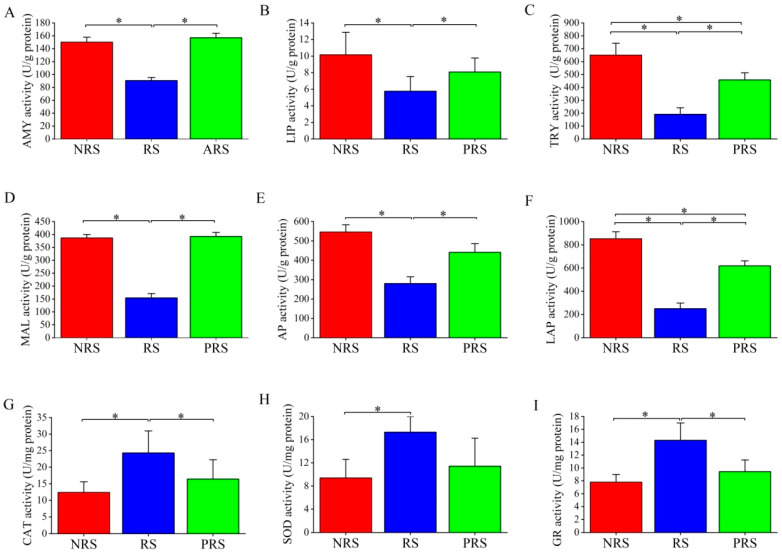
The digestive enzymes activities (**A**–**F**) and antioxidant capability (**G**–**I**) of intestinal tract of the black Amur bream in the different reproductive statuses (NRS. non-reproductive stage; RS. reproductive stage; PRS. post-reproductive stage); (**A**). Amylase (AMY); (**B**). Lipase (LIP); (**C**). Trypsin (TRY); (**D**). Maltase (MAL); (**E**). Alkaline Phosphatase (AP); (**F**). Leucine–alanine peptidase (LAP) (**G**). Catalase (CAT); (**H**). Superoxide dismutase (SOD); (**I**). Glutathione reductase (GR). Note: Values in the table are represented as Mean ± SE, *n* = 8; “*” notes significant difference among different groups, *p* < 0.05.

The distribution of the biological parameters across different groups is shown in Table 1. There was no significant difference in SL among the three groups (*p* > 0.05). Wt, GSI, ED, K, and Lip were at higher levels in the RS group than in NRS and PRS groups, whereas RGL, FD, and Wat showed the opposite pattern. Pro showed an increasing trend from the NRS to the PRS groups. Histological observations of the *M. terminalis* ovary across different groups are displayed in Figure 1A–C. Remarkable variation in oocytes was observed in relation to the reproductive stage. Oocytes were present in groups NRS, CA, Vtg1, and Vtg2, while group RS showed late GVM oocytes. Group PRS ovaries exhibited large numbers of POFs and a few CA and vitellogenic (Vtg1, Vtg2) oocytes. Furthermore, the intestinal mucosa of fish samples in the RS group showed a certain degree of damage and hyperemia compared with the NRS and PRS groups (Figure 1D–F). The intestinal epithelium of *M. terminalis* displayed an abundance of mucous cells. AB-PAS staining showed that intestinal mucous cells could be divided into four types (I–IV) (Figure 1G–I). The NRS and PRS mucous cells were largely type I and III mucous cells, while the RS had more type II and IV mucous cells. As shown in Table 2, the density of type II and IV mucous cells in the RS group was significantly higher than in the NRS and PRS groups (*p* < 0.05). In contrast, the density of type III mucous cells in the NRS was higher than those in the RS and PRS groups (*p* < 0.05). The total density of mucous cells in the RS group was the lowest among all groups. The number of eosinophilic granulocytes in the intestinal submucosa of RS samples was significantly higher than in the NRS and PRS groups (Figure 1J–L, Table 2). In contrast, the number of mitochondria in the intestinal epithelial cells of RS samples was much lower than those in the NRS and PRS groups (Figure 1M–O, Table 2). Mitochondria in intestinal epithelial cells of NRS and PRS samples had a complete structure with inner ridges visible, while mitochondria in the intestinal epithelial cells of RS showed signs of apparent autophagy (Figure 1M–O). The intestinal digestive and antioxidant enzyme activity of *M. terminalis* is shown in Figure 2. The digestive enzyme activity in the RS group was much lower than in the NRS and PRS groups (*p* < 0.05) (Figure 2A–F). The activities of trypsin and aminopeptidase enzymes in the PRS group were significantly lower than that of NRS (*p* < 0.05). However, antioxidant enzyme activity exhibited the opposite pattern (Figure 2G–I). CAT, SOD, and GR activities of RS gut samples were much higher than in NRS samples, and the CAT and GR activity of the PRS group was lower than that of the RS group (*p* < 0.05).

### 2.2. Gut Microbiota Diversity and Ecological Networks among the Non-Reproductive, Reproductive and Post-Reproductive Groups

**Figure 3:**
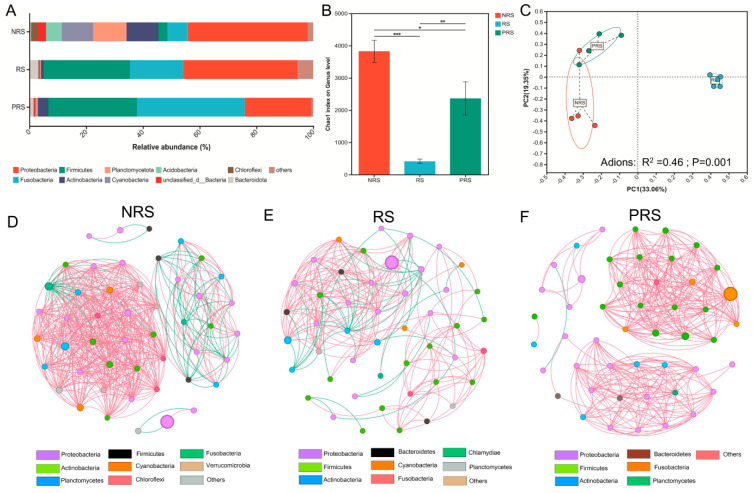
An overview of the data. (**A**) A bar graph illustrating the comparative abundance of various phyla within the dominant phylum across distinct categories of fish guts. Only those with a mean relative abundance exceeding 1% within the phylum are displayed. “Unclassified” designations were applied to sequences that could not be assigned to a phylum. (**B**) Alpha diversity results of the gut microbial community for three groups distributed in the various reproductive statuses of *M. terminalis* are presented; * *p* < 0.05, ** *p* < 0.01, and *** *p* < 0.001 denote statistical significance, respectively. (**C**) The PCoA ordination and adonis test in the *M. terminalis* metagenomes (999 permutations with adjusted *p*−value) using Bray–Curtis distance matrices for bacterial species−based metagenomes; (**D**–**F**) co-occurrence networks of gut microbiota among three groups distributed across the various reproductive stages of *M. terminalis*. The analyses were conducted utilizing the top 50 most abundant genera of the intestinal microbial community across the various groups. The significance level of the data presented in the figure was *p* < 0.05, with r ≥ 0.6. The abundance is proportional to the size of the circle representing a species. The correlation between two genera is symbolized by the line’s hue, with red denoting a positive correlation and green representing a negative correlation. The intensity of the correlation is indicated by the thickness of the edge. Note: NRS. non-reproductive stage; RS. reproductive stage; PRS. post-reproductive stage.

The gut microbiota of the three distinct reproductive groups exhibited significant variations. The gut bacteria of NRS and RS were mostly Proteobacteria at the phylum level, accounting for 44.2% and 68.1%, respectively. The gut bacteria of PRS were characterized by Fusobacteria (40.3%) and Firmicutes (30.9%) indicated clear differences (Figure 3A). The alpha diversity (Chao1) of the NRS and PRS groups was much higher than that of the RS group at the genus level (Figure 3B). In addition, we generated a PCoA analysis based on Bray–Curtis distance from the gut microbial communities (species level) that showed that the samples in the RS groups were obviously separated from the NRS and PRS groups (Adions: *R*^2^ = 046; *p* = 0.001; Figure 3C).

Co-occurrence networks generated based the on significantly correlated genus (with Spearman coefficients *r* > 0.6 and *p* < 0.5; Figure 3D–F) revealed potential changes in microbial interactions among the three groups. Co-occurrence network analyses showed that the correlations exhibited different patterns among the three groups, with more connections in microbial communities from the NRS group (475 edges, positive 85.1% and negative 14.9%) and PRS (326 edges, positive 99.7% and negative 0.03%) compared to the RS group (216 edges, positive 79.2% and negative 21.8%) (Figure 3D–F). The average degree value, which indicates network complexity, was significantly greater in NRS (9.87) and PRS (6.77) compared to RS (4.57) based on the computed network topological properties.

**Figure 4:**
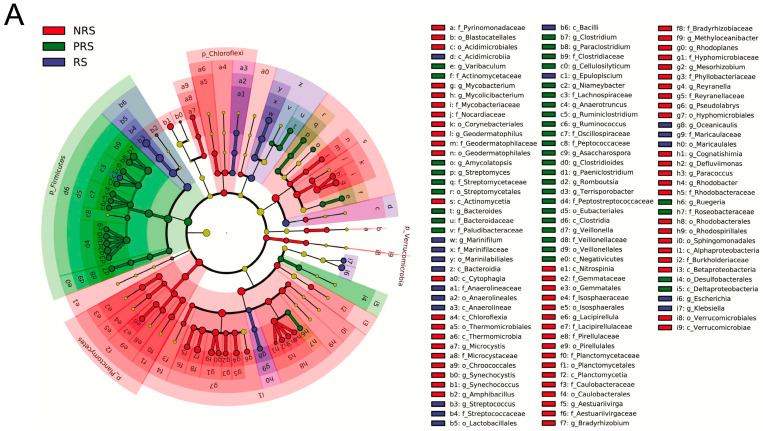

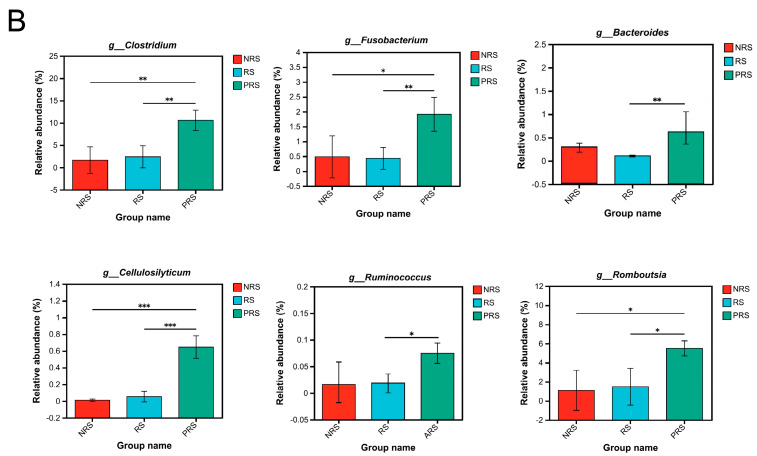
Putative gut microbial response to the reproductive behavior of the black Amur bream. (**A**) Using linear discriminant analysis effect size, the significance difference (*p* < 0.05) in the relative abundance of abundant taxa among three distinct groups was ascertained; (**B**) the relative abundance of six species of gastrointestinal microbes that are implicated in the metabolism of carbohydrates in each fish group. For pairwise comparisons, the Kruskal–Wallis test was utilized. The significance level of a variable is denoted by * *p* < 0.05, ** *p* < 0.01, *** *p* < 0.001.

In addition, the LEfSe analysis revealed several microbial taxa that were significantly enriched in the various groups, as shown in Figure 4A. The consensus characteristic seen in the gut microbiota was a high enrichment of the phylum Firmicutes in the PRS samples (Figure 4A). For example, *Clostridium*, *Romboutsia*, *Fusobacterium*, and *Paeniclostridium* could be considered as microbiological markers to differentiate the PRS group from the other groups (Figure 4A). On the other hand, we found that phylum Proteobacteria in the NRS group presented much higher LDA scores than those in the other groups (Figure 4A). Fewer microbial taxa were identified as significantly enriched in the RS groups. The LDA scores of the genera *Marinifulum*, *Streptococcus*, *Oceanicaulis, Escherichia,* and *Klebsiella* were significantly higher in the RS group than in other groups (Figure 4A).

**Figure 5:**
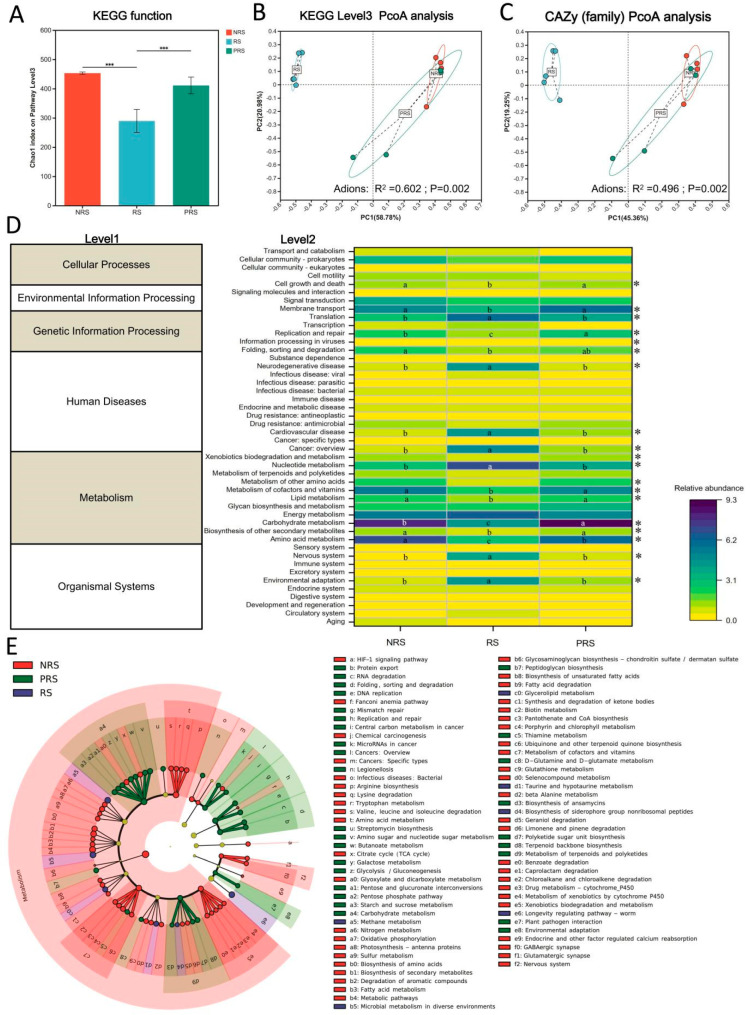
Gut microbiota community function differences in the non-reproductive, reproductive and post−reproductive groups. (**A**) The alpha−diversity divergence (Chao1 index) as determined using KEGG function classification was displayed in boxplots. The significance level of a variable is denoted by *** *p* < 0.001. Differences in the beta diversity associated with the nonreproductive, reproductive, and post−reproductive groups as determined using KEGG level 3 (**B**) and CAZy (family) (**C**). The Bray–Curtis distance matrices were utilized in the PCoA ordination and adonis test (at 999 permutations with an amended *p*−value) on the *M. terminalis* metagenomes, which were determined using the KEGG function at level 3 and CAZy families. (**D**) KEGG categories obtained via PICRUSt2 from the metagenome of fish intestine microbiomes. The relative abundance of gut bacterial gene functions (level 2) across the three distinct groups is depicted as a heatmap. Samples denoted with an asterisk (*) among four categories signify significant differences (*p* < 0.05). Different capital letters denote significant differences among four categories (a > b > c; *p* < 0.05) for samples designated with such letters. (**E**) LEfSe identified the KEGG pathways that were significantly enriched in the non-reproductive, reproductive, and post-reproductive groups. LDA scores greater than four and *p* < 0.05 are displayed. NRS stands for non-reproductive stage; PRS denotes the post-reproductive stage.

Further investigation focused on six genera (*Clostrium*, *Fusobacterium*, *Bacteroides*, *Cellilosilyticum*, *Ruminococcus*, *Romboutsia*) closely associated the carbohydrate metabolism indicated that the PRS group had a significantly higher relative abundance compared to the RS group (Kruskal–Wallis test, *p* < 0.05; Figure 5B). Moreover, only the relative abundance of *Clostrium*, *Fusobacterium*, *Cellilosilyticum* of PRS samples was significantly higher in the NRS samples (Figure 4B).

### 2.3. Gut Microbiota Functional Differences among Non-Reproductive, Reproductive and Post-Reproductive Groups

In the current study, we assessed the functional diversity of the gut microbiome in three groups using the Chao1 index and KEGG functional gene annotation at level 3. The Chao1 index was significantly lower in the RS group compared to the NRS and PRS groups (Kruskal–Wallis test, *p* < 0.001; Figure 5A). Differences in the KEGG functional pathways (level 4) of the gut microbiota were specifically noted between the RS group and the NRS and PRS groups (Adions *R*^2^ = 0.602, *p* = 0.002; Figure 5B). Similarly, a difference in carbohydrate-active enzyme (CAZyme) function categories was noted across three groups (Adions *R*^2^ = 0.496 and *p* = 0.002; Figure 5C).

Moreover, a total of 18 KEGG pathways (level 2) exhibited significant differences in abundance among the three groups, e.g., carbohydrate metabolism, amino acid metabolism, lipid metabolism, nervous system, and environmental adaptation (Figure 5D). Carbohydrates, amino acids, lipids, and energy metabolism were enriched in the PRS and NRS groups, while nervous system, environmental adaption, and nucleotide metabolism were enriched in the RS group (*p* < 0.05).

**Figure 6:**
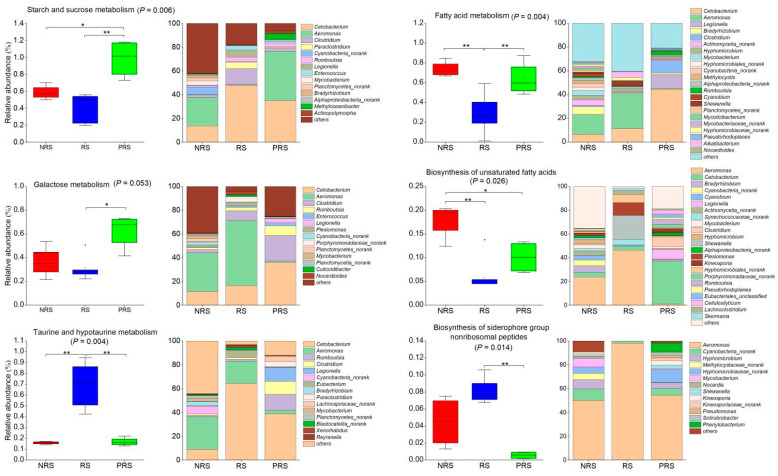
The relative abundance and bacterial source of the target KEGG pathways associated with reproductive adaptation of the black Amur bream. For pairwise comparisons, the Kruskal–Wallis test was utilized. The significance level of a variable is denoted by * *p* < 0.05, or ** *p* < 0.01. NRS stands for non-reproductive stage; PRS denotes the post-reproductive stage.

Metagenomic research utilizing KEGG revealed the possible involvement of the *M. terminalis* gut microbiome in responding to changes during the reproductive life cycle. The LEfSe analysis indicated that the KEGG pathways enriched in each group were highlighted in the cladogram (*p* < 0.05; Figure 5E). The relative abundance of starch and sucrose and the galactose metabolism were enriched in the PRS samples, whereas the relative abundance of fatty metabolism and biosynthesis of unsaturated fatty acids were enriched in the NRS samples (Figure 6). Moreover, taurine and hypotaurine metabolism and the biosynthesis of siderophore group nonribosomal peptides was also consistently enriched in the RS gut metagenomes (Figure 6).

## 3. Discussion

The investigation found notable variations in biological indices across various groups, particularly in GSI, FD, K, and Lip. RS individuals had a greater body size and higher GSI compared to NRS and PRS individuals, as seen in the southern flounder research [23]. Lipid levels in the black Amur bream’s ovaries rose throughout the reproductive phases and decreased post-reproduction. Lipids are acknowledged as high-energy compounds used for energy storage in fish [24,25]. Recent research has shown that fish use lipids to control their energy consumption for gonad growth [24]. Our prior research showed significant differences in the lipid levels of the ovary, serving as a key physiological indicator of breeding readiness in *M. terminalis* [22]. Interestingly, we found that the FD of RS individuals was at a very low level. This result was similar to the pattern observed in many fish species, where the fish are at a low feeding level during the process of gonad maturation to spawning [26]. It has been proved that the hormone changes in the fish during the reproductive period may be one of the reasons for the decreasing of feeding intensity [27]. On the other hand, fish metabolic prioritization has a close relationship with lifestyle and habitat (fast flowing vs. slow flowing) [20]. In our previous study, the stimulation of flood peak is regarded as a key factor for the breeding success of *M. terminalis* [21]. Notably, RS *M. terminalis* is faced with a more fast-flowing habitat compared to NRS and PRS individuals. Fish inhabiting fast-flowing habitats typically attain their aerobic metabolic ceiling, also known as their maximal metabolic rate, when exposed to physical activity [20]. Therefore, it can be inferred that *M. terminalis* potentially demonstrates a compromise between two metabolic types: one associated with locomotion, which seems to have diminished digestive capabilities but improved locomotor performance, and the other associated with visceral function, which facilitates digestion throughout the various reproductive phases.

Based on the above assumption, we further tested fish gut morphological and physiological features in individuals at different reproductive stages. The mucous cells in the intestine of RS individuals were generally acidic, and the numbers of neutral mucous cells were significantly reduced, resulting in the loss of the function nutrient absorption [28,29]. Relevant research has examined the acid mucous cells distributed in the digestive tract of fish containing various mucosaccharides and immunoglobulins, proteins involved in fish immunity, while neutral mucous substances can coexist with alkaline phosphatase and have the function of regulating the pH of the digestive tract and thereby assisting in digestion [30]. Mitochondria in intestinal epithelial cells of reproductive individuals also showed a similar autophagy phenomenon (Figure 1E). When cells are under stress due to reactive oxygen species, nutrient deficiency, and senescence, the autophagy mechanism recognizes and deletes the damaged mitochondria in order to regulate the number of mitochondria and maintain cellular energy metabolism [31]. Furthermore, comparing the activities of digestive enzymes in the different groups, the digestive enzyme activities in the intestines of reproductive *M. terminalis* were lower than those in the other groups (Figure 2). It seems likely that the NRS and PRS *M. terminalis* have the strongest ability for digestion and absorption of nutrients, while the RS *M. terminalis* have a weaker ability to digest food. Recent research has revealed that the activities of amylase, trypsin, and lipase were positively correlated with the absorption of various nutrients that are important indicators of digestive physiology [32]. Moreover, alkaline phosphatase and leucine aminopeptidase mainly exist in shallow fish intestinal epithelial cells with striated borders, functioning to help intestinal epithelial cells absorb metal enzymes [19].

Our results indicated intestinal microbial diversity significantly decreased in RS individuals compared to NRS individuals and PRS individuals. The decline of microbial diversity observed in RS individuals may be closely related to the decreased feeding level. It has been reported that microbial diversity, as a key factor in the stability of the gut microbiome, can be influenced by various host selection pressures, e.g., physiological behaviors such as feeding and reproduction [12,29,33]. Relevant research indicated that host dietary changes can contribute to significant alterations of the gut ecosystem [29]. Additionally, we discovered that the gut microbiota of fish individuals differed significantly at each reproductive stage (Figure 3C). The interaction between host reproductive hormones and gut microorganisms has been shown to have substantial impacts on the host metabolism throughout the mating period, according to recent research [10,34]. For instance, reproductive hormones, especially progesterone, in female Phayre langur monkeys (*Trachypithecus phayrei*) contribute to changes in gut microbiota during pregnancy and lactation [35]. Moreover, our results showed that many competitive interactions were in the network of gut microbiota in the RS *M. terminalis*, whereas cooperative interactions dominated the network of gut microbiota in the PRS *M. terminalis* (Figure 3E,F). Microbial interactions (from mutualism to competition or symbiosis) are critical for shaping the gut microbiota composition and function [36,37]. The little interspecific competition among gut bacteria leads to the stability of the host’s gut microbiota [36]. Studies have shown that maintaining the stability of the microbial structure in fish guts may enhance physiological function and facilitate the exploitation of new ecological niches [7].

Our previous findings illustrated that the reproductive behavior of *M. terminalis* consumed a large quantity of energy [22]. However, we found that the FD of RS individuals was still at a very low level. Meanwhile, the intestinal microbial diversity of RS was also low. In general, wildlife describes a decline in the diversity of its intestinal microbiota as a result of diminished dietary diversity [38]. As mentioned in our previous points, the breeding process of *M. terminalis* requires energy for reproduction on the one hand [22]. On the other hand, reproductive *M. terminalis* requires energy to swim in a fast-flowing habitat [21]. We observed a rapid decline in intestinal cell energy metabolism, digestive enzyme activity and gut microbiota diversity, which is due to the energy metabolic-type tradeoff of reproductive samples. *M. terminali* depleted the body of stored energy substances (e.g., fat) by reducing the level of food intake. Lipids are essential energy sources with excellent energy efficiency in the somatic tissues of fish [27]. *M. terminalis* needs metabolism to sacrifice its own digestion and improve the level of swim metabolism in order to improve its reproductive success rate.

After the breeding period, *M. terminalis* usually consume a large amount of food to restore physical strength [7]. At the end of the reproductive phase, the relative abundances of *Clostridium Fusobacterium*, *Bacteroides*, and *Cellulosilyticum* associated with carbohydrate metabolism are significantly enriched in PRS samples (Figure 4B). Carbohydrates are well known as efficient energy materials for animals. Highly idiosyncratic responses to diet are strongly associated with different gut microbiota [39]. Simultaneously, we observed that the number of neutral mucous cells in the intestinal mucosa layer of PRS samples also significantly increased (Table 2). The mucus layer of the host gut can adhere to and protect the microorganisms inhabiting the gut, subsequently making them viscoelastic in the intestinal environment [40,41]. Furthermore, neutral mucin, as one of the components of the mucous layer, can be degraded by some microorganisms to provide carbon sources. The digestive enzyme activity of PRS groups also sharply increased compared to the RS groups, verifying the recovery of fish intestinal digestive function [19]. Complex interactions between changes in physiological function and fish gut microbes play important roles in stabilizing and maintaining the ability of the microbiota to respond to perturbations. In addition, our investigation of pathogenic bacteria among three different groups showed that *Aeromonas veronii* and *Strptococcus dsygalactiae* were enriched in the RS gut content samples (Supplementary Figure S1). Meanwhile, our results showed that the number of acidic mucous cells and eosinophilic granulocyte saw an obvious increase in the RS group, and the antioxidant enzyme activity of the host gut was also significantly up-regulated. These physiological changes may be considered as feedback from the significant increase in the abundance of intestinal pathogens [42]. Secreting acidic mucus in the intestinal epithelium can reduce the pH in the intestinal cavity to inhibit the growth of harmful bacteria, thereby protecting the intestine from invasion by pH-sensitive pathogens [29,41]. During the reproductive process of *M. terminali*, harmful bacteria invaded the host and caused the host immune response.

Additionally, we observed great KEGG and CAZy function divergence between the RS and NRS and PRS groups. Our results also showed a greater functional variety and an increase in functional genes related to carbohydrate, lipid, amino acid, cofactors, and vitamin metabolic pathways in the NRS and PRS group. The results indicated that variations in the reproductive state of a host may alter the natural composition and functional capabilities of its gut microbiome. This enables animals to cultivate distinct gut microbiomes to adjust to significant changes in surroundings or diets during their reproductive life cycle. In the present research, we further focused on six important functional pathways. The genes encoding the putative enzymes involved in fatty acid metabolism and the biosynthesis of unsaturated fatty acids were enriched in the NRS samples (Figure 6). Before spawning, *M. terminali* tends to consume foods of high-nutrient foods (e.g., high protein and fat) in order to store enough energy in the body for reproductive behavior [12,22]. It is noteworthy that an enrichment of genes encoding putative enzymes implicated in the metabolism of taurine and hypotaurine was observed in the RS samples. Taurine and hypotaurine have been documented to perform significant functions in animals, including the promotion of motor ability and mitochondrial health [43,44]. Therefore, we deduced that the gut microbiota putatively responds to the host’s fast-flowing environment. In our study, *Cetobacterium* and *Aerominas* played an essential role in taurine and hypotaurine metabolism in the gut of the RS population. The genes responsible for enzymes related to starch, sucrose, and galactose metabolism in the PRS group were significantly increased, indicating a potential energy compensation mechanism in *M. terminali* following breeding. *Cetobacterium* and *Clostridium* are of great importance in starch and sucrose and galactose metabolism in the gut of the PRS population. The PRS group has greater microbiome diversity and composition compared to the RS group, indicating a varied and complex community of microbial fermenters capable of breaking down a wide range of feed for digestion. In this study, the interaction between the gut microbiome and *M. terminalis* at different reproductive stages was necessary to maintain a dynamic balance, and when the stability of the healthy state is perturbed, an unhealthy stable state would eventually occur [45,46].

## 4. Materials and Methods

### 4.1. Fish Sampling

**Figure 7:**
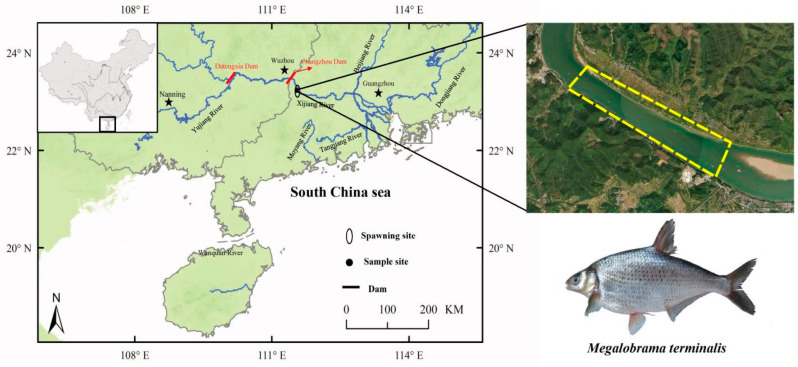
Schematic representation of the sampling sites for the black Amur bream. Stars indicated City.

In this study, fifty-five *M. terminalis* individuals were gathered from the Luopan spawning area in the main stem of the Pearl River using circular cast nets (16 m diameter, mesh size 3 cm) between 1 July and 15 July 2021 (Figure 7). Measurements of water temperatures, salinity, dissolved oxygen (DO), and pH at sampling sites were conducted using an HQ30 device (Hach Company, Loveland, CO, USA). The results are presented in Supplementary Table S1. Standard length (SL) is the distance from the tip of the mandible to the base of the caudal fin. Body weight (Wt), eviscerated weight (EW), and gonad weight (GW) were measured to the nearest 1 g and 0.01 g, respectively. Gut length (GL) was measured to the nearest 1 mm and relative gut length (RGL) was calculated as the ratio of gut length to body length. The feeding intensity was determined by the filling degree (FD) of the intestinal tract, the average filling degree, the feeding rate, and the jejunum rate. The filling degree of the intestinal tract was assigned according to Table 3 [47]. The reproductive stage of *M. terminalis* was identified for all individuals based on the morphological and histological characteristics, as described by previous study [22]: non-reproductive stage (NRS) (*n* = 20), reproductive stage (RS) (*n* = 17), post-reproductive stage (PRS) (*n* = 18). The gonadosomatic index (GSI = 100 × GW/EW) was estimated as an indicator of the fish reproductive period, and fatness (K = 100 × Wt/SL^3^) was measured as bioenergetic indices to evaluate fish conditions.

### 4.2. Biochemical Assays

In order to conduct the biochemical analysis of the fish, ten specimens were dissected from each group. Left ovary sections were extracted from each fish, which were then placed in ice-sealed plastic containers and frozen at −80 °C until processing. Each sampled fish’s tissue was homogenized and freeze-dried at −80 °C for 24 h to a constant weight prior to analysis. Upon cooling and weighing the resulting dry tissue, the water content (Wat) was calculated as (100–% dry tissue). The crude protein (Pro) content was determined using the Kjeldahl method [48], whereas the crude Lipid (Lip) content was assessed using the chloroform–methanol extraction method [49].

### 4.3. Histological and Ultrastructural Investigations

#### 4.3.1. Light Microscopy

Anesthesia was administered to eight samples of *M. terminalis*, each with a distinct reproductive status, using MS-222 (0.2 g L^−1^ MS-222 + 0.2 g L^−1^ NaHCO_3_). The samples were subsequently dazed and rapidly killed by being decapitated. For histological analysis, the left ovaries and intestines of the fish were dissected out and fixated with Bouin’s fixative for 24 h. After embedding the tissues in paraffin wax, sections measuring 5 mm in thickness were obtained and subjected to staining using Alcian blue-Periodate Scheff (AB-PAS) and hematoxylin-eosin (H&E). The Alcian Blue-PAS (AB-PAS) staining technique divides the mucus cells of the large-scaled loach into four types: Type I, labeled in red, is AB-negative and PAS-positive, indicating the presence of neutral mucopolysaccharides. Type II, labeled in blue, is AB-positive and PAS-negative, indicating the presence of acidic mucopolysaccharides. Type III, labeled in purple-red, shows positive reactions for both AB and PAS, primarily indicating the presence of PAS-positive neutral mucopolysaccharides. Type IV, labeled in blue-purple, shows positive reactions for both AB and PAS, primarily indicating the presence of AB-positive acidic mucopolysaccharides [28].

#### 4.3.2. Transmission Electron Microscopy

Each group’s intestinal tissue underwent the following procedures: pre-fixation with 2.5% glutaraldehyde, three washes with phosphate-buffered saline (PBS), fixation with 1% osmic acid, dehydration via ethanol gradient, acetone clearance, epoxy agent 650 polymer embedding, and RMC ultramicrotome sectioning to a thickness of 70 nm [50]. Uranyl acetate and lead citrate were utilized to double-stain the ultra-thin sections, which were then observed through an H-7800 transchromatic electron microscope (TEM).

#### 4.3.3. Morphometric Measurements

The densities of mucous cells, mitochondria in intestinal epithelial cells, and eosinophilic granulocytes in submucosa were quantified using ImageJ. Three slides were made for each intestinal segment of *M. terminalis*. Ten fields were randomly chosen for observation, resulting in thirty measurements per fish sample.

### 4.4. Enzyme Assays

We chose eight samples from various reproductive stages of *M. terminalis*. The frozen samples were taken out of the refrigerator (deep frozen at −80 °C) and put on ice to defrost. After being thawed, the gut samples were mixed thoroughly on ice in 0.2 M NaCl using an F6/10 Fluko homogenizer at 12,000× *g* for 2 min. The homogenate was centrifuged using a cryogenic ultracentrifuge, and the supernatant was used to assess the activities of digestive enzymes and soluble proteins. All the enzymatic activity (including Amylase, AMY; Lipase, LIP; Trypsin, TRY; Maltase, MAL; Alkaline Phosphatase, AP; Leucine–alanine peptidase, LAP; Catalase, CAT; Superoxide dismutase, SOD; Glutathione reductase, GR) was quantified using an enzyme test kit (NO: C016, A080-2, A054-2, A080-1, A059-2, H454, A007, A001, A062, Nanjing Jiancheng Bioengineering Institute, Nanjing, China). The particular activity was quantified as units per gram of protein or units per milligram of protein (mU/mg protein or U/mg protein). The concentration of soluble protein (mg/mL) was measured using the Bradford technique using bovine serum albumin as the standard (0.563 g/L) [51]. The tests were conducted on triplicate samples using an Infinite M200 Pro Tecan Sunrise (Tecan, Hombrechtikon, Switzerland). Corning 96-well microplates from Corning Incorporated, which are UV-permeable, were used for all experiments.

### 4.5. Metagenomic Analysis

#### 4.5.1. Fish Gut Contents Collection

To eliminate transient microorganisms and prevent skin surface contamination, sterile instruments were utilized to dissect the entire digestive system of a solitary fish sample. Following this, the sample was immediately cleansed in a solution of 75% ethanol and sterile water. For the purpose of sequencing, samples were randomly combined from three groups containing four or five gut contents. The specimens were promptly immersed in liquid nitrogen, subsequently transferred to an ultra-low temperature freezer, and maintained at −80 °C until further use.

#### 4.5.2. DNA Extraction, Library Construction, and Metagenomic Sequencing

CTAB (cetyltrimethylammonium bromide) was utilized to extract total genomic DNA from fish samples [52]. The quantification of the extracted DNA’s concentration and purity was performed utilizing a NanoDrop2000 and a TBS-380, respectively. The purity of the DNA extracts was evaluated by means of 1% agarose electrophoresis. In order to generate paired-end libraries, DNA samples were fragmented to an average size of 400 base pairs using a Covaris M220 instrument manufactured. By utilizing the NEXTFLEX Rapid DNA-Seq reagent (Bioo Scientific in Austin, TX, USA), the paired-end library was generated. Ligation was used to affix adapters containing hybridization sites for all sequencing primers to the distal ends of fragments. The paired-end sequencing procedure was executed on an Illumina NovaSeq platform manufactured by Majorbio Bio-Pharm Technology Co., Ltd. in Shanghai, China. NovaSeq Reagent Kits were utilized in accordance with the manufacturer’s instructions.

#### 4.5.3. Sequence Quality Control and Genome Assembly

Fastp version 0.20.0 was utilized to truncate the paired-end Illumina reads in order to eradicate adaptors and low-quality reads (length < 50 bp, quality value < 20, or containing N bases) [53]. The location where the tool can be accessed is https://github.com/OpenGene/fastp accessed on 18 May 2024. Using BWA (version 0.7.9a, available at http://bio-bwa.sourceforge.net accessed on 18 May 2024) [54], the reads were aligned to the host genome. Hits that were also linked to the paired reads were omitted from the alignment. The compilation of the metagenomic data was conducted utilizing MEGAHIT version 1.1.2, an application that employs succinct de Bruijn graphs [55]. The repository hosting the software is https://github.com/voutcn/megahit accessed on 18 May 2024. As the final assembly outcomes, contigs with a minimum length of 300 base pairs were selected and employed for subsequent gene prediction and annotation.

#### 4.5.4. Gene Prediction, Taxonomy, and Functional Annotation

The prediction of open reading frames (ORFs) was performed for every assembled contig utilizing Prodigal v2.6.3 (https://github.com/hyattpd/Prodigal accessed on 18 May 2024). The amino acid sequences of the predicted ORFs with a length of at least 100 base pairs were obtained and converted to their respective forms using the NCBI translation table (http://www.ncbi.nlm.nih.gov/Taxonomy/taxonomyhome.html accessed on 18 May 2024). CD-HIT (version 4.6.1, http://www.bioinformatics.org/cd-hit/ accessed on 18 May 2024) was utilized to generate a nonredundant gene catalog with 90% sequence identity and 90% coverage [56]. In order to compute gene abundance with a 95% confidence level, high-quality sequences were aligned to the nonredundant gene catalogs utilizing SOAPaligner (version 2.21; https://bio.tools/soap accessed on 18 May 2024). For subsequent analysis, we exclusively retained the unigenes that belonged to bacteria, and we obtained the taxonomic information for these bacterial unigenes. For taxonomic annotations, representative sequences of nonredundant gene catalogs were aligned to the NR database using Diamond (https://github.com/bbuchfink/diamond accessed on 18 May 2024, version 0.8.35) with an e-value threshold of 1e-5 [57]. Following this, taxonomic data were gathered regarding these bacterial unigenes. The unigenes underwent functional annotation using the CAZy and Kyoto Encyclopedia of Genes and Genomes (KEGG) databases [58].

#### 4.5.5. Bioinformatic Analysis

The gut microbiota data underwent statistical analysis using the R (version 3.2.1) package vegan for diversity analysis, PCoA, Adions, and Procrustes analysis [59]. The study used linear discriminant analysis effect size (LEfSe) to identify notable variations in the proportion of gut microbiota and KEGG pathways across non-reproductive, reproductive, and post-reproductive samples, aiming to pinpoint microbiome biomarkers [60]. A co-occurrence network analysis was performed using the Networkx module in Python. Network visualization and modular analysis were conducted using Gephi version 1.10.1. The potential genes in the specified KEGG pathway were compared to the NCBI NR database using the diamond software (version 4.6) We identified the potential microbiome origins of these KEGG pathways in each metagenome. We compared the discovered genes with the Pathogen Host Interactions Database available at http://www.phi-base.org/index.jsp accessed on 18 May 2024.

### 4.6. Statistical Analysis

One-way ANOVA in SPSS Statistics 28.0 was used to assess variations across groups in biological parameters (SL, Wt, GSI, K, RGL, and FD), proportions of biochemical constituents (Pro, Lip, and Wat), activities of digesting enzymes, antioxidant capacity, and alpha diversity. The normality of the data and homogeneity of variance were assessed with the Kolmogorov–Smirnov test and Levene’s test, respectively. The study used a Kruskal–Wallis test to examine variations among the three experimental groups. ANOVA and Kruskal–Wallis tests were used for multiple group comparisons, followed by Tukey’s post hoc test unless stated otherwise. Statistical significance is shown by * *p* < 0.05, ** *p* < 0.01, and *** *p* < 0.001. Significance tests for bacterial community composition were conducted using Adonis analysis with 999 permutations in the vegan package for R. The study was performed on categories selected significantly based on Bray–Curtis distances [61].

## 5. Conclusions

In this study, we discovered a potential link between metabolic-type tradeoff and the changes in gut microbiota of *M. terminalis* across different reproductive statuses. The influence of choosing metabolic types throughout different reproductive phases on the nutritional approach and digestive function of fish was significant. Our research reveals how the host’s metabolic type and reproductive state might impact the structure, function, and metabolic activities of the gut microbiome. We found that the feeding behavior significantly influences the gut microbiota of reproductive *M. terminalis* more than non-reproductive and post-reproductive samples. The study showed that the stability and resilience of the gut bacterial community in wild fish may be influenced by interactions between the host and microbiome. The intestinal physiological characteristic was shown to have a significant role in determining the functional composition of gut microbiomes. However, it is necessary to clarify how the altered gut microbiota influences host behavior and physiology, which themselves enhance the host’s reproductive success, in a future study.

## Figures and Tables

**Table 1:** Basic biological and biochemical information of female *M. terminalis* in the different groups.

	Item	NRS	RS	PRS
biological information	SL (mm)	254 ± 31 (20)	263 ± 21(17)	257 ± 26 (18)
	Wt (g)	371.6 ± 26.9 (20) ^b^	420.6 ± 35.4 (17) ^a^	350.6 ± 33.7 (18) ^b^
	GSI (%)	2.35 ± 0.38 (20) ^b^	10.59 ± 2.14 (17) ^a^	4.31 ± 1.97(18) ^b^
	K	2.41 ± 0.35 (20) ^b^	2.62 ± 0.28 (17) ^a^	2.35 ± 0.31 (18) ^b^
	RGL	3.51 ± 0.23 (20) ^a^	3.21 ± 0.54 (17) ^b^	3.52 ± 0.42 (18) ^a^
	FD	3.75 ± 0.15(20^) b^	0.65 ± 0.12 (17) ^c^	4.25 ± 0.25 (18) ^a^
biochemical constituent proportion of ovary	Pro (%)	15.31 ± 2.14 (10)	13.08 ± 3.01(10)	11.35 ± 1.96 (10)
	Lip (%)	11.93 ± 1.84 (10) ^ab^	15.88 ± 2.94 (10) ^a^	9.58 ± 1.47 (10) ^b^
	Wat (%)	68.25 ± 3.65 (10) ^ab^	63.56 ± 4.15 (10) ^b^	73.57 ± 5.11 (10) ^a^

Note: SL, standard length; Wt, body weight; GSI, gonadosomatic index; K, fatness; RGL, relative gut length; FD, fullness degree; Pro, protein; Lip, lipid; Wat, water. Different superscript letters indicate significant differences in component in the different groups, a > b > c; *p* < 0.05.

**Table 2:** Morphometric measurements of intestinal tract of female *M. terminalis* in the different groups.

Item		NRS	RS	PRS
Mucous cell	Type I	1.27 ± 0.21 ^a^	0.51 ± 0.23 ^b^	1.16 ± 0.31 ^a^
	Type II	0.89 ± 0.14 ^b^	2.18 ± 0.37 ^a^	0.61 ± 0.11 ^c^
	Type III	8.75 ± 1.19 ^a^	2.02 ± 0.34 ^c^	5.75 ± 0.75 ^b^
	Type IV	3.68 ± 0.75 ^b^	5.82 ± 1.05 ^a^	3.95 ± 0.93 ^b^
	Total	14.59 ± 2.27 ^a^	10.53 ± 1.94 ^b^	11.47 ± 1.87 ^ab^
Mitochondria in intestinal epithelial cells		5.71 ± 1.07 ^a^	2.14 ± 0.83 ^b^	6.92 ± 1.32 ^a^
Eosinophilic granulocyte in intestinal submucosa		0.45 ± 0.33 ^b^	3.86 ± 0.93 ^a^	0.79 ± 0.56 ^b^

Note: Values in the table are represented as Mean ± SD of mucous cell number in 100 μm × 100 μm; values in the table are represented as Mean ± SD of mitochondria number in intestinal epithelial cells in 1 μm × 1 μm; values in the table are represented as Mean ± SD of eosinophilic granulocyte number in intestinal submucosa in 10 μm × 10 μm; the data with different superscripts in the same row are significantly different (a > b > c; *p* < 0.05).

**Table 3:** Judgment of gut fullness.

Fullness	Description
0	Jejunum or a very small amount of food was observed in the intestinal tract
1	Only part of the bowel has a small amount of food or a quarter of the bowel is food
2	There is only a small amount of food or half of the food in the entire bowel
3	More food, medium filling degree, food accounted for 3/4 of the bowel
4	It is a lot of food. It is filling up the intestines
5	There was too much food, and the intestines swelled

## Data Availability

Metagenome sequencing data and relevant files have been uploaded to Sequence Read Archive with the accession number PRJNA 1061225.

## Supplementary Materials

The supporting information can be downloaded at: https://www.mdpi.com/article/10.3390/ijms25137425/s1.

## Abbreviations

Abbreviation	Full title
AMY	Amylase
AP	Alkaline Phosphatase
CAT	Catalase
FD	Fullness degree
GR	Glutathione reductase
GSI	Gonadosomatic index
GVM	Germinal vesicle migration
K	Fatness
LAP	Leucine–alanine peptidase
LIP	Lipase
Lip	Lipid content
MAL	Maltase
NRS	Non-reproductive stage
POF	Post-ovulatory follicle
Pro	Protein content
PRS	Post-reproductive stage
RGL	Relative gut length
RS	Reproductive stage
SL	Standard length
SOD	Superoxide dismutase
TRY	Trypsin
Vtg1	Primary vitellogenic oocyte
Vtg2	Secondary vitellogenic oocyte
Wat	Water content
Wt	Body weight
YG	Yolk
