# Supplementary material for: The Response of the Gut Physiological Function and Microbiome of a Wild Freshwater Fish (Megalobrama terminalis) to Alterations in Reproductive Behavior
Source: Int J Mol Sci. 2024 Jul 6;25(13):7425. doi: 10.3390/ijms25137425 (PMC11242598; doi:10.3390/ijms25137425)
Supplement: Supplementary file 1 [file ijms-25-07425-s001.zip › ijms-3040900-supplementary.pdf]

SUPPLEMENTARY MATERIALS

Table S1 Selected environmental characteristics for the sampling sites of *M. terminalis*

Environmental Parameter	Quantitative value
Temperature (℃)	26.9~28.7
Salinity (‰)	0.008~0.01
pH	7.9~8.3
DO (mg/L)	6.9~7.1

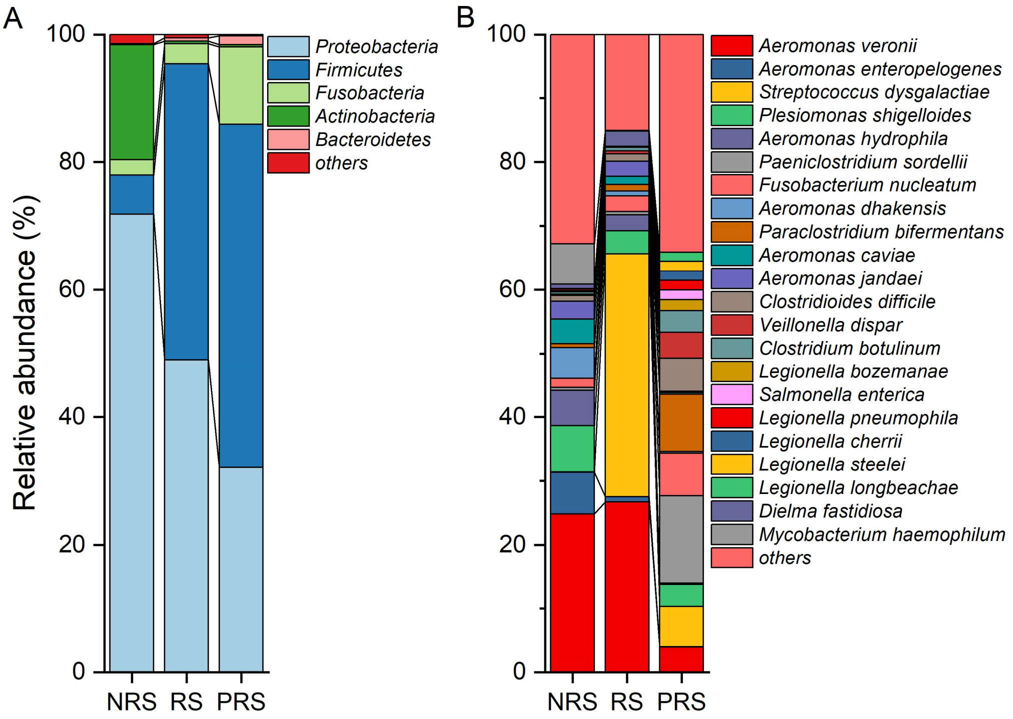

Figure. S1 Bar plot presenting the relative abundance of different pathogenic phyla and species in different fish gut groups. Only those with mean relative abundance more than 5% for phylum and species are shown.
